# Factors Influencing Professional Help-Seeking Behavior During First Episode Psychosis in Schizophrenia: An Exploratory Study on Caregivers’ Perspective

**DOI:** 10.3389/fpsyt.2019.00962

**Published:** 2020-02-14

**Authors:** Daniel Teck Lung Wong, Seng Fah Tong, Tuti Iryani Mohd Daud, Salina Abdul Aziz, Marhani Midin

**Affiliations:** ^1^ Department of Psychiatry and Mental Health, Faculty of Medicine, Universiti Kebangsaan Malaysia Medical Centre, Kuala Lumpur, Malaysia; ^2^ Department of Family Medicine, Faculty of Medicine, Universiti Kebangsaan Malaysia Medical Centre, Kuala Lumpur, Malaysia; ^3^ Department of Psychiatry and Mental Health, Hospital Kuala Lumpur, Kementerian Kesihatan, Kuala Lumpur, Malaysia

**Keywords:** schizophrenia, first episode psychosis, factor, help-seeking behavior, qualitative study, Malaysia

## Abstract

Schizophrenia is a severe mental illness that leads to significant productivity loss and is listed in the top 15 global burdens of disease. One important contributor to the high disease burden is duration of untreated psychosis (DUP) which can be shortened with promotion of professional help-seeking behavior. This study explored caregivers' perspective on factors influencing professional help-seeking behavior during first episode psychosis (FEP) in schizophrenia in Malaysia. The results of this study would inform the development of intervention strategies targeted at promoting professional help-seeking behavior in caregivers of individuals experiencing first episode psychosis (FEP). This is a thematic exploratory study which employed purposive sampling using focus group discussion (FGD). These interviews were audio recorded and transcribed verbatim. Basic thematic approach was used in analyzing the transcribed interviews. Two main themes identified were adequacy of knowledge and stigma. These two factors were found to co-influence each other. Stigma undermined the impact of knowledge on professional help-seeking; likewise, the reverse was also observed. Intervention strategies for promoting help-seeking behavior during FEP should simultaneously focus on improving knowledge about schizophrenia and reducing the stigma attached to it.

## Introduction

Schizophrenia is one of the leading contributors to the high disease burden caused by mental, neurological and substance-use (MNS) disorders. It contributes to 16.8 million of disability-adjusted life year (DALY) (1) and remains one of the top 15 leading causes of disability worldwide (2). In Malaysia, productivity loss resulting from Schizophrenia amounts to USD 100 million a year (3). Despite the high burdens caused by the illness, research to improve its outcome is still inadequate. In addressing the huge research gap in mental health, the Global Mental Health Initiative has identified priorities for research to improve the lives of people with mental illness worldwide (1). A total of 6 major goals encompassing 25 specific challenges has been listed (1). Goal B which is “Advance prevention and implementation of early interventions”, targets at reducing the duration of untreated illness by developing culturally-sensitive early interventions (1).

Duration of untreated psychosis (DUP) is one of the factors contributing to the high disease burden in schizophrenia. Meta-analyses studies show a consistent correlation between long DUP and poor outcome (4, 5) Patients with schizophrenia who have a longer DUP prior to initial treatment have poorer symptom control and impaired long-term social functioning (6, 7), necessitating higher cost for their treatment and care. In Malaysia, mean DUP for schizophrenia is as long as 37.6 months (8), which is a huge concern in terms of the potential poor outcome and high burdens the illness brings to the individuals, families and the country.

The impact of DUP on illness outcome has led researchers to focus on first episode psychosis (FEP) in the last two decades. Many studies have been done to understand the barriers and facilitators of professional treatment-seeking that contribute to DUP and at the same time determine the pathways to care for people with FEP. Among the socio-demographic factors, education level has been found to be correlated with DUP while others such as age, gender, marital status, living situation and professional status had no evidence of association with DUP (7, 9–11). Cannabis use, which has a role in precipitating schizophrenia (12), was not found to influence DUP (9). Individual temperament and the availability of social support were found to play a role in DUP, whereby, good social support and low neuroticism helped to reduce DUP (13). Delayed treatment can also be due to under identification by general practitioners of some insidious features of the emerging psychosis (13).

Previous qualitative studies on pathways to care of patients with FEP indicated that that help-seeking behavior was affected by stigma, lack of symptom awareness, tendency to normalize psychotic symptoms, and lack of correct knowledge about treatment services (14–16). Support from significant others and availability of relevant information, on the other hand, were highlighted in those studies as important facilitating factors (14–17). For example, Cadario et al. found that many young people with FEP relied on others to access help (17) and Boydell et al. highlighted that help seeking as a social process that involved persuasive influence of significant others (15). Understanding this process of help-seeking behavior in FEP is crucial to inform development of relevant early interventions. In countries such as Australia, advancement the in understanding of many aspects FEP through research has contributed to the development of systematic delivery of evidence-based early interventions for this group of people (18, 19).

Research in FEP is far lagging behind in the Asian countries as compared to the western countries. Research findings from the western countries may not be applicable to the lower-resourced and culturally diverse Asian countries. Several studies done in India, both quantitative and qualitative, have investigated factors affecting help-seeking behavior in schizophrenia (20–22). These studies indicated the presence of unique cultural factors as determinants of help-seeking (21) which may be different in Malaysia with its unique multi-racial population. In Malaysia, studies done in this area have used checklists and interviewer directed questionnaires (23, 24) which may have missed some important details not covered by these quantitative data-collection tools. Qualitative method which encourages free-flow expression of experiences is a better option to explore the rich help-seeking-related information that have influenced care-givers' and patients' decisions to come forward to seek professional treatment.

We conducted a study to explore caregivers' perspective on factors influencing professional help-seeking behavior during FEP in schizophrenia patients as part of developing a comprehensive and appropriate educational tool to promote earlier psychiatric help-seeking behavior in Malaysia.

## Materials and Methods

### Study Design and Setting

This study is a thematic exploratory study which employed a qualitative generic inductive approach using Caeli’s generic principles (25). Such design allows better insight into the caregivers’ perspectives on important information related to professional help-seeking behavior. The study was conducted from September 2018 till November 2018 on caregivers of schizophrenia patients at Universiti Kebangsaan Malaysia Medical Centre (UKMMC) in Kuala Lumpur, Malaysia. We have adopted COREQ Guidelines (26) in reporting.

### Recruitment and Sampling

The study employed a purposive sampling method. A spectrum of participants was selected to represent different stages of recovery from schizophrenia, different educational levels and the major ethnicities in Malaysia. Purposive sampling is effective in identifying and selecting information-rich cases in settings with limited resources (27).

Inclusion criteria were family caregivers who were; 1) having a family member fulfilling diagnostic criteria of DSM-5 for Schizophrenia; 2) 18 years old or older; 3) being the main caregiver for the patient since the onset of the illness and; 4) able to converse in Malay or English language. Exclusion criteria were caregivers; 1) with concurrent life threatening medical condition or cognitive impairment and; 2) caregivers with the ill family member being in acute psychotic episode or facing life threatening medical condition or having dual diagnoses.

### Data Collection and Data Analysis

Data collection was done via focus group discussion (FGD) consisting of an average of four participants. FGD sessions were initially co-conducted by first author and two senior co-researchers who are experienced in qualitative study and schizophrenia. All facilitators were introduced to participants as doctors, either an academic psychiatrist, psychiatrist in training or academic family physician. We emphasized that FGD was a session where researchers attempt to understand caregivers’ experiences in making their way to seek professional help during the time when their ill family members had FEP. Independent sessions were conducted by first author subsequently when his interview technique was deemed satisfactory.

FGD was chosen over In-Depth Interview (IDI) as caregivers were anticipated to be able to share their experiences in a closed group. Furthermore, FGD would help group members retrieve memories of their experiences when others share theirs and promote empowerment among participants to voice their needs for information related to help-seeking and treatment of this highly stigmatized illness.

FGDs were conducted by using semi-structured facilitator guide (**Table 1**). The topic guide consisted of open-ended questions with in-depth probing to allow a thorough understanding about the objectives in their own words to gain rich qualitative data. Duration of discussions ranged from 1 hour 10 minutes to 2.5 hours. All FGDs were audio-recorded with digital audio device and then transcribed verbatim.

**Table 1:** Semi-structure interview guide.

Semi-structured Interview Guide
What information had stopped the caregivers from engaging psychiatric health care service?
What factors had discouraged them to seek professional (psychiatry) help?
What information had brought them to psychiatric health care
What factors had motivated them to seek professional (psychiatry) help

Data were analyzed using thematic analysis after each data collection session to identify and generate major themes and sub-themes. We adopted basic descriptive thematic analysis suggested by Braun and Clarke (28). We aimed at providing overarching descriptive themes from the data collected and propose how these themes influence caregivers help-seeking behaviors during FEP in patients with schizophrenia. Transcripts were read first to gain an overall understanding. This was followed by initial coding and theme identification. Themes were constructed by comparing and contrasting the initial codes. Data analysis was done by the first author. To improve confirmability of data analysis, independent analysis was undertaken by two senior co-researchers with experience in qualitative study on two randomly chosen transcripts. In addition, analysis meetings were held with experienced co-researchers to discuss the coding framework, emerging themes and to interpret analysis. After each data analysis session, the questions for subsequent sessions were refined before proceeding with subsequent data collection. This research involved reading the data repeatedly to familiarize with the data and manually generating codes. The codes were reviewed, re-categorized and rearranged to clearly define a theme. Data analysis was done manually. QSR NVivo 12 Computer-Assisted Qualitative Data Analysis Software was used to ease organization of data.

The cycle of participant recruitment, data collection and analysis was continued until data saturation was achieved. Data saturation is reached when new data tend to be repeating of data already collected (29). Descriptive analytic report was generated from the themes in relation to the objectives.

## Results

### Socio-Demographic of Participants

A total of 18 (n = 18) primary caregivers of patients with schizophrenia who fulfilled the study criteria were interviewed in five focus group discussions (**Table 2**). They were aged between 33 to 69 years; 8 male and 10 female. Among them, 7 were Malay, 10 Chinese, and 1 Indian. Duration of care ranged from 5 to 20 years. The participants' relationship to the person with schizophrenia were either father (n = 5), mother (n = 6), spouse (n = 2), sister (n = 4) or brother (n = 1). Most of the participants (n = 16) were married, with 2 others either widowed or divorced. Ten were employed, 1 retired, and 7 unemployed.

**Table 2:** Socio-demographic data of participants (n = 18).

Focus group	Participant	Gender	Age (year)	Race	Duration of care (years)	Relationship with patient	Marital status	Employment
1	Par A	F	53	C	9	Mother	Married	E
	Par B	F	69	M	5	Mother	Married	UE
	Par C	M	63	M	9	Father	Widower	UE
	Par D	F	51	M	7	Mother	Married	UE
2	Par E	M	67	C	20	Father	Married	E
	Par F	M	44	C	20	Brother	Married	E
	Par G	F	54	M	6	Mother	Married	UE
	Par H	F	61	I	29	Sister	Married	Retired
3	Par I	M	40	C	8	Husband	Married	E
	Par J	F	58	M	20	Sister	Married	E
	Par K	F	62	C	20	Mother	Married	UE
	Par L	F	42	C	15	Sister	Divorced	E
	Par M	M	63	M	10	Father	Married	UE
4	Par N	M	53	M	9	Father	Married	E
	Par O	F	50	C	5	Mother	Married	UE
	Par P	M	52	C	5	Father	Married	E
5	Par Q	M	43	C	7	Husband	Married	E
	Par R	F	33	C	10	Sister	Married	E

Par, participant; M, Malay; C, Chinese; I, Indian; E, employed; UE, unemployed.

Factors influencing help-seeking behavior during FEP are listed in **Table 3**.

**Table 3:** Factors influencing professional help-seeking during FEP.

Themes	Sub-themes
Adequacy of Knowledge	Knowledge on how schizophrenia could present itself Knowledge on effectiveness of psychiatric treatment for schizophrenia Attribution of schizophrenia symptoms to non-schizophrenic illness Attribution of cause of psychotic behaviour Knowledge on treatment setting Knowledge on resources to facilitate help-seeking
Stigma	Internal stigma External stigma

### Theme 1: Adequacy of Knowledge

#### Knowledge on How Schizophrenia Could Present Itself

Schizophrenia presented in a spectrum of different symptom variety and severity. Inadequate knowledge on the different types of presentation especially the subtle symptoms tended to delay caregivers' detection of FEP. Caregivers either overlooked or regarded it as normal variation of psychological experiences or responses to social stressors.

“… I don't realize she has symptoms because ah … I, I thought after her big examination, she just wanted to relax, so she kept herself in the room…” (Participant C, 2018)

“…he couldn't work, he told my dad he couldn't work. So, we thought that he was just giving excuses, being lazy” (Par L 2018)

Another participant shared inadequate knowledge on certain behavioral changes being symptoms of schizophrenia. This participant’s son stopped playing badminton and suddenly went missing during training (patient was in badminton team of his school and was involved in an intensive training program at the onset of illness). The patient’s subsequent behavior of being socially withdrawn and keeping himself in his room was seen by the caregiver as a normal adolescent behavior which had substantially delayed professional help-seeking behavior.

“…I don't know whether it's because of pressure … he refused to play badminton and then went missing, suddenly missing from training. He was then found sitting in the hall at night. At that time, I didn't know that he was ill. I just didn't know about it. I thought that, ‘Aiyoo, this must be a young man's attitude problem. So, it's okay if you (referring to his son) do not want anything' … so he did not come out (paused for 3 seconds) you know, for a year in the room…” (Participant E, 2018)

It was common among the caregivers that awareness about the mental illness only began when prominent aggressive behavior set in. One participant shared about his awareness that his wife was ill and needed treatment only when she demonstrated aggressive behavior.

“So, ah, so how I end up to (to bring my wife) this hospital is ah, ah, it was in a private [sic] place, in a coffee shop, she behaved insanely, very serious, argue and she also try to damage people's ah, stuff lah that time. Then I know at that time that she must be having illness and not normal, not just the usual suspicious…” (Participant Q, 2018)

Besides aggressive behavior, prominent hallucinatory behavior and severe deterioration in functionality were other behavioral changes that triggered caregivers' awareness of the state of unwellness in the patients which prompted them to seek treatment. For example,

“….form 3 beginning of the year okay, middle of the year, about June like that, he talking, he talk with the teacher first, he say hear the voice talking to him, then we thought, nothing one lah, normal one lah, sometime, heard people talking, heard people talking, like that also what. He talk to the teacher, teacher also listen what he says lah, come back he also, saw something there, we ask us come and see there got people talking there, talk about him, then we go and see, eh, nothing one, nothing one we also don't bother lah. We thought he was just being naughty. Then more and more, the thing is serious already until one day, around that PMR (Penilaian Menengah Rendah - form 3 public exam in Malaysia), ah PMR right, before the PMR got one exam one, oh he doesn't study, and in the exam he go and take out the book and find answer, answer the question. Then the teacher said, eh, something wrong already. So, during that time, we only started to realize he is unwell and need treatment.” (Participant O, 2018)

Some caregivers could pick up behavioral changes as abnormal but had no idea they could be treated medically as they had never heard or seen anyone with a similar problem.

“For me, not because I don't want to try, because I don't know about modern medication, this sort of abnormal behavior can be treated in the hospital, I don't have that knowledge … If I have the knowledge, I will come earlier, I can do both, I can go medical I can go traditional.” (Participant C, 2018)

“maybe the TV should play a role also. Instead of every now and then, educating people that Aedes mosquitoes has white stripes, they should also put and explain err how to recognize the symptoms of mental illness, if this person has such and such behavior for instance, don't leave it, ah help him, tell us what should be done, help him. because most of us don't know the symptoms of schizo, how to help if we don't what is schizo?” (Participant B, 2018)

Participants reported they would have brought the patients for treatment earlier if they knew the changes in the patients were symptoms of schizophrenia. This indicates knowledge about symptoms of schizophrenia would facilitate professional help-seeking behavior among caregivers of patients experiencing FEP.

“Yes, yes definitely, if I am a doctor with knowledge, if I see my son having symptoms of schizophrenia, then I will definitely bring my son to hospital straight away … Because from 11 years old, sigh (paused for 2 seconds) if we know that is the symptom of schizophrenia, immediately we go… (Participant N, 2018)

If we knew the symptoms we would straight away go and see the doctor.

(Participant O, 2018)

#### Knowledge on Effectiveness of Psychiatric Treatment for Schizophrenia

Knowledge about the effectiveness of psychiatric treatment had motivated the caregivers to seek treatment for the patients under their care. Some obtained the information from personal experience of being a carer for another schizophrenia patient in the family. Meanwhile, others obtained such information from friends or people around them.

For instance, one participant gained her knowledge about the effectiveness of psychiatric treatment from her experience in caring for another family member with schizophrenia.

“… my mother-in-law had this illness (schizophrenia) also, she was ill after she delivered my husband. My mother-in-law receive real treatment (psychiatric treatment) lah for her schizo … she recovered with treatment (psychiatric treatment).” (Participant G, 2018)

The same participant went on to equip herself through reading about schizophrenia before her daughter presented with symptoms of schizophrenia.

“… I researched (reading articles related to schizophrenia) because my mother-in-law had it (schizophrenia)… before my daughter had it, I had, had, had already read about this mental illness (schizophrenia)…” (Participant G, 2018)

Another participant came to know about psychiatric treatment effectiveness from a friend.

“Ya, ya, ya, because ah, my friend's father also had similar condition (Schizophrenia), he (friend) told that this must be because of that (Schizophrenia) lah. He say that his father is now okay after hospital treatment.” (Participant E, 2018)

A few participants gained knowledge of psychiatric treatment benefits through the hard way after attempts with traditional treatments failed to improve the patient's condition which prompted them to switch to psychiatric treatment. Subsequently they opted to continue psychiatric care after they witnessed its effectiveness. For example, participant C brought his daughter for psychiatric treatment when she showed little improvement even after five years of religious therapy.

“Well, I say okay, why don't give it a try, because we have done it (referring to religious therapy) for 5 years you see, without any improvement. So okay, I accept the advice why not? It's just a try. This is one of the efforts for us too, you know, bring her to treatment lah (referring to psychiatric treatment). Then it's okay. It's a good suggestion actually. Not, not many people know about this. That's why you can see there are still ah … a lot of untreated people…” (Participant C, 2018)

#### Attribution of Schizophrenia Symptoms to Non-Schizophrenic Illness

In some cases, caregivers knew that patients were unwell. However, wrong attribution of symptoms of schizophrenia by themselves or the primary care doctors had delayed the initial psychiatric treatment.

“I have a friend who have a autistic son, err … Down syndrome. So I was thinking ah, I, I don't know err … what kind of mental illness my son is having. So my, my friend having a son, a Down syndrome, so I was thinking ah Darren is more or less like that, mental illness maybe another type, and I don't know what type, because at that time I'm a…. I don't do much reading about this mental illness, so I don't know much.” (Participant A, 2018)

The same participant consulted a doctor who misled him by giving a wrong diagnosis.

“I brought him to see a doctor in Pudu (pause 2 seconds) after taking some medication, the doctor was telling me that he was autistic.” (Participant A, 2018)

Another participant who knew something was wrong with his son and was ready to accept treatment, was assured by a doctor that treatment was unnecessary and what his son needed was just rest.

“Ah … I, I, I did bring him to see private. The private doctor says, because he is, ah … he is a … what we call … ah … too much of pressure. So, just ask him to relax.” (Participant E, 2018)

#### Attribution of Cause of Psychotic Behavior

From this study, we found that different attributions of illness influenced the types of treatment sought. For example, one carer initially brought his ill family member for spiritual treatment as he believed the behavior changes in his son were caused by spiritual factors. Psychiatric treatment was not sought until he was enlightened by his friend who worked in a hospital on psychiatric illness being a potential explanation for these behavioral changes.

“When he come back, we still think, it is ah something to do with ah, spiritual. So, we take him to the Ustad (religious teacher) for the treatment … And we have a relative, who happens to be a somebody in hospital … and he recommended to bring him to see the psychiatrist as he explained to us that my son may have psychiatric illness, so we met Dr Susan.” (Participant N, 2018)

Participants who did not believe in traditional treatment were open to suggestion for medical treatment. For example:

“I don't actually think about that lah (traditional method), I don't think so. It's because I enter into Uni and I am a so called ‘science' student, herbs yes I believe is one of the cure, but that is not a long term solution and I study this before, I know this is because of the neurotransmitter, so, I don't think any herbs or spiritual treatment can actually improve it. So, when my uncle suggested to bring my brother to hospital for treatment, I agreed. (Participant R, 2018)

#### Knowledge on Treatment Setting

Caregivers became reluctant to bring patients for psychiatric treatment when they were misinformed about unfavorable psychiatric treatment setting.

“… initially when the policeman advised me to send my son to general hospital, I refused. I thought that my child will be sent to Tanjung Rambutan (a mental institute in Malaysia), so I don't want … I am afraid because people there are naked and fight with each other, so I am worried and consider for so long … I got to know about it from media when I was so much younger, when I was in my twenties. I came across paper which talked about happening in Tanjung Rambutan for two, three days … so I thought that all psychiatric treatment are scary…” (Participant A, 2018)

Subsequently, caregivers were encouraged to bring the patients for treatment when they eventually found out that the psychiatric treatment setting could be conducive. A participant made a personal visit to a psychiatric clinic before deciding to bring his son for treatment.

“Before I brought Darren here, I came to psychiatric GH (psychiatric clinic in general hospital) alone. I looked and walked around the clinic. I asked the patient, ‘what happened during doctor's consultation? Any abuse going on?' I went on and spoke to [a] few patients and mothers, then I found out that the hospital environment is not bad … Yes. I really regretted, I should come earlier, it's such a great place, all the nurses, all the doctors they are so understanding…” (Participant A, 2018)

#### Knowledge on Resources to Facilitate Help-Seeking

Some caregivers were aware of mental illness as a potential explanation for the behavioral changes in the patients. They were aware of the availability of modern treatment; however they struggled to bring the patients to the hospital due to patients' refusal. They were informed that police can be involved only in the event of aggression. Therefore, they had to wait until aggression set in.

“No, not try, we actually wanted to do that (bring patient to hospital) for a long time. But my brother refused to come to hospital, I even call the hospital ambulance myself, and ask and I even ask does anyone from hospital can do us a favor, send him to the hospital to get treatment? The hospital say they cannot force if patient refuses … Then they said if really don't want to come to treatment or what, you have to call police and ask the police to bring. So, the only way to get him I thought is the police. That's the only way I know. But police say if the patient is not aggressive, they also cannot do anything… 10 years, after 10 years he only get proper treatment.” (Participant L, 2018)

Many caregivers were unaware of the availability of community psychiatric services that could facilitate access to psychiatric treatment.

Participant J

“You aware of that (referring to community psychiatry service)? are you aware? I'm not aware.”

Participant L

“Oh, you are not aware? I am not aware of it too, but I call around and ask, I call and ask if they have a team to support my brother, my brother doesn't even want to go to the hospital … finally I got to know about community psychiatry service…” (Participant J & L, 2018)

### Theme 2: Stigma

Stigma was found to be an important factor in influencing help-seeking behavior among the caregivers of patients with schizophrenia in this study. A number of caregivers felt disgraced by the psychotic behavior of schizophrenia patients due to internalized prejudices related to mental illness.

“I [was] ashamed to share with my friends, only some close ones. Initially I'm ashamed you know. I [was] ashamed to let people know … that my son is different, so … I only let 2 good friends know, the rest of my friends, I will never tell. And, because he always creates havoc for me ah, function I won't bring him, I will bring my other son. Because he really gives me … a lot of havoc. I am worried that my friend will look down upon me for not teaching my son properly.” (Participant A, 2018)

“… none of my husband's family knew about it (referring to her daughter who is diagnosed with schizophrenia). Ah, because (paused for 3 seconds) don't know lah … my husband also didn't allow me to, didn't allow me to share with his siblings. He felt shameful, worried that he will be look[ed] down by others.” (Participant G, 2018)

Besides prejudices, some caregivers had internalized stereotypic beliefs about mental illness.

“Family want to keep to themselves because *malu lah* (feeling shameful), something is wrong with the gene is it?” (Participant J, 2018)

Social stigma was another factor which had delayed professional help-seeking behavior in this study. Some caregivers perceived help-seeking as involving exposing the patients with schizophrenia to others, and that it would lead to disrespect and isolation from the local community.

“Actually, in our community, accepting a mentally ill patient is indeed, indeed negative lah. We felt, we felt that as if we are marginalized. It is not exactly the feeling of being insulted but felt more or less like that. That's why as long as we can hide it (about one of the family members being mentally ill), we hide it…” (Participant M, 2018)

### Relationship Between Adequacy of Knowledge and Level of Stigma

**Figure 1:**
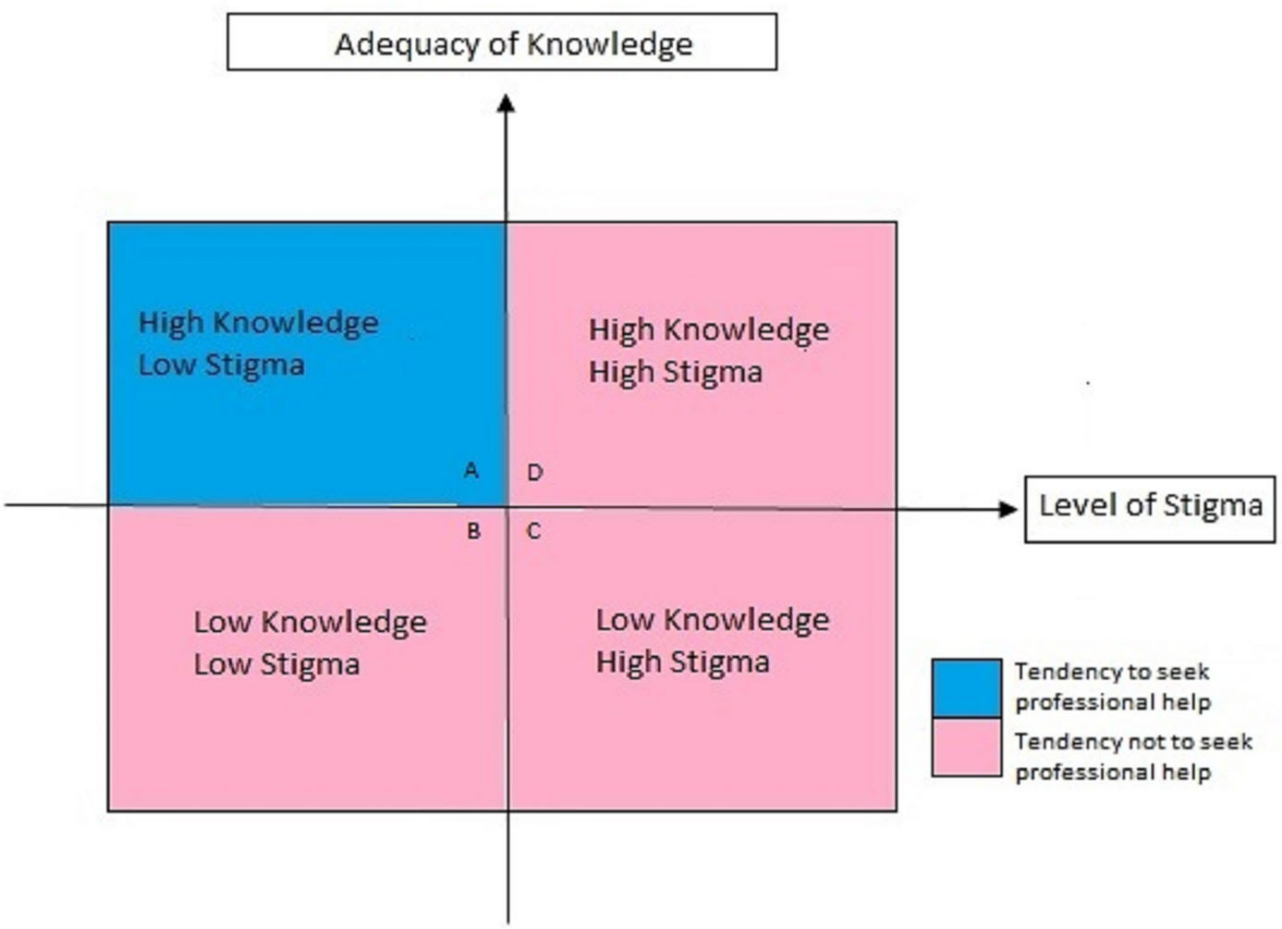
Dynamic relationship of knowledge and stigma.

In this study, two main domains of factors that influenced professional help-seeking were identified: adequacy of knowledge and level of stigma. These two factors can be seen as a matrix of dynamic interplay between them in influencing professional help-seeking behavior, as illustrated in **Figure 1**. In this matrix, the dynamic interplay between adequacy of knowledge and level of stigma can be divided into four different quadrants, which is best illustrated through examples from the participants.

#### (Quadrant A) High Knowledge, Low Stigma (High Tendency in Seeking Professional Help)

The presence of adequate knowledge coupled with low level of stigma tends to facilitate professional help-seeking. Participant E, who did not have much stigma toward psychiatric treatment, brought his son to a hospital the moment he gained the knowledge about mental illness and availability of effective treatment.

“… I did not prejudice psychiatrist. All types of doctors are the same, must consult doctor if there's an illness … Ya, ya, ya, because ah, my friend's father also had similar condition (Schizophrenia), he (friend) told that this must be because of that (Schizophrenia) lah. He say that his father is now okay after hospital treatment. Only then I knew that my son might be ill as well, so I immediately brought him (referring to son) to consult Psychiatrist in University Hospital…” (Participant E, 2018)

#### (Quadrant B) Low Knowledge, Low Stigma (Low Tendency in Seeking Professional Help)

Individuals tend not to seek professional help when knowledge about mental illness is lacking, even though level of stigma is low. The same participant in the previous example, i.e. Participant E, who did not have much stigma toward psychiatric treatment, experienced a substantial delay in seeking professional help for his son due to lack of knowledge about mental illness. He described himself as somebody who did not think negatively about psychiatrists at the onset of his son's illness.

“…I don't know whether it's because of pressure or not … he refused to play badminton and then went missing, suddenly missing from training. He was then found sitting in the hall at night. At that time, I didn't know that he was ill. I just didn't know about it. I thought that, ‘Aiyoo, this must be a young man's attitude problem. So, it's okay if you (referring to his son) do not want anything'. So, he did not come out (paused for 3 seconds) you know, for a year in the room … no, no, I did not prejudice psychiatrist. All types of doctors are the same, must consult doctor if there's an illness. I didn't know that he was already ill when he started to isolate himself…” (Participant E, 2018)

#### (Quadrant C) Low Knowledge, High Stigma (Low Tendency in Seeking Professional Help)

Lower tendency in seeking professional help is seen when an individual has inadequate knowledge and high stigma, as illustrated by participant A's experience. Participant A who lacked adequate knowledge about schizophrenia and psychiatric treatment was exposed to a media report on aggressive incidents occurring in a mental hospital. This negative image about mental hospitals spread fast among the local community which shaped her view about psychiatric treatment. This biased information from the media (inadequate information), contributed to her stereotypic belief (stigma) about all psychiatric treatment settings being horrible and psychiatric patients being aggressive. She started to discriminate **(stigma)** psychiatric treatment since then.

“As what I mentioned earlier on, I was twenty plus that time, I read from [the] newspaper about people in Tanjung Rambutan was hurt, can't remember exactly but is something about patients fighting in there. Scary-lah when I read the news, like like doctors just leave the patients alone-lah, patient fighting lah, naked lah, hitting lah, so scary. Many people also talk about it, very very hot topic that time. My friends that time also describe all the scary things to me … Ya, since then I always thought that psychiatric treatment is bad, is something more or less like that lah … I don't want psychiatric treatment, I worried that my son will be treated like that lah (referring to unfavorable psychiatric treatment).” (Participant A, 2018)

#### (Quadrant D) High Knowledge, High Stigma (Low Professional Help-Seeking Behavior)

High level of knowledge about schizophrenia is not equivalent to high tendency of seeking professional help if stigma level remains high. This dynamic interplay is illustrated through the experience of participant A. Participant A who had negative views toward psychiatric treatment since her early adulthood was very reluctant to seek psychiatric treatment when her son started to develop schizophrenia. She approached the police for help once when her son became aggressive and was informed by a police officer that her son was probably having mental illness and required psychiatric treatment. Increase in knowledge on mental illness in her did not facilitate professional help-seeking due to her remaining stereotypic belief about unfavorable psychiatric treatment in hospitals which led to a significant delay in seeking psychiatric treatment. Therefore, improvement in knowledge about treatment availability without reducing stigma is inadequate to promote help-seeking behavior.

“…my friend has a son, a Down syndrome. So, I was thinking ah D (initial of son's name) is having something more or less like that.” (Participant A, 2018).

“The police are the one who advised me to bring my son to come to hospital. He said, ‘… please go to hospital, this is mental illness, it can be treated. There are other people who had similar conditions like this, but they are now well after treatment.'…” (Participant A, 2018)

“… initially when the policeman advised me to send my son to general hospital, I refused. I thought that my child will be sent to Tanjung Rambutan (a mental institute in Malaysia), so I don't want … I am afraid because people there are naked and fight with each other, so I am worried and consider for so long … I got to know about it from media when I was so much younger, when I was in my twenties. I came across paper which talked about happening in Tanjung Rambutan for two, three days … so I thought that all psychiatric treatment are scary…” (Participant A, 2018)

She finally brought her son to hospital when her level of stigma reduced after seeing for herself that psychiatric treatment setting is not necessarily unpleasant.

“Before I brought Darren here, I came to psychiatric GH (psychiatric clinic in general hospital) alone. I looked and walked around the clinic. I asked the patient, ‘what happened during doctor's consultation? Any abuse going on?' I went on and spoke to [a] few patients and mothers, then I found out that the hospital environment is not bad … Yes. I really regretted, I should come earlier, it's such a great place, all the nurses, all the doctors they are so understanding…” (Participant A, 2018)

The two examples from the experiences of Participant A and E, illustrate the dynamic interplay of knowledge and stigma. Level of both factors changed over time when these participants went through different life experiences and were exposed to relevant information. An individual can be in different quadrant of the matrix at a different point in time and move to another quadrant at another time. From this study, it can be concluded that tendency to seek professional help is higher when an individual has high level of knowledge about schizophrenia and, at the same time, low level of stigma toward it.

## Discussion

In this study, factors influencing psychiatric help-seeking behavior during FEP among caregivers of patients with schizophrenia were explored *via* qualitative approach. A diverse set of factors that influenced decision to first psychiatric treatment were identified and can be broadly classified into adequacy of knowledge and level of stigma. This finding is consistent with findings from previous studies on factors influencing professional help-seeking behavior in schizophrenia during FEP (14–17, 20–22, 30). The study findings also shed some light on how these factors are closely linked and influence each other in determining professional help-seeking behavior.

In this study, various aspects of knowledge about schizophrenia was found to contribute to professional help-seeking behavior among caregivers of patients with schizophrenia during their FEP (from knowledge on symptoms to information about and how to seek treatment). In terms of knowledge on symptoms of schizophrenia, without adequate knowledge on how the illness might present itself, subtle symptoms tended to be overlooked and not recognized by caregivers in this study. Many caregivers normalized such subtle changes which eventually delayed professional help-seeking. These findings are not surprising and have been described in other studies (15, 16, 21). In a qualitative study looking at how people identify and respond to emerging psychosis, Judge et al. (16) found themes like normalization, giving explanation, withdrawal, avoiding help and coming to terms with psychosis which posed as barriers to help-seeking (16). On the other hand, obvious illness presentation such as aggressive and disorganized behavior had facilitated awareness of potential mental illness in some caregivers in this study, hence prompting professional help-seeking. This is consistent with findings from another local study using quantitative method (23).

Interestingly, we found that obvious symptoms did not necessarily motivate professional help-seeking behavior in caregivers. Caregivers in this current study experienced other barriers to professional help-seeking related to knowledge about schizophrenia. Firstly, attribution of cause of psychotic behavior was observed to play role as a barrier in help-seeking. Those who attributed psychotic behavior to magico-religious cause had opted for spiritual remedy over psychiatric treatment as their initial approach. This finding was also observed in other Asian studies (20, 23). Professional help-seeking behavior was hence delayed despite the caregivers having successfully identified the presentation of schizophrenia. In contrast, caregivers who attributed psychotic behavior to scientific causes were more open to suggestion for medical treatment. This finding suggests that cultural and religious factors can be important determinants of help-seeking in schizophrenia and therefore should be incorporated in the development of educational materials aimed to improve knowledge about schizophrenia.

Secondly, a few caregivers who were ready to seek medical treatment faced barriers at the general practitioner (GP) level in this study. In these cases, the GP offered inaccurate attribution of schizophrenia symptoms to non-schizophrenic illness such as ADHD or psychological stress, hence delaying psychiatric treatment. Under-diagnosis and misdiagnosis of specific mental disorders are common among GPs, and this could be improved when symptoms presented are clearly reflecting psychotic conditions and being raised explicitly to the GPs (31, 32). This gap of knowledge at different levels were closely linked and influenced each other but at the same time, they could be a standalone factor that affects professional help-seeking behavior. As the old saying goes, the eyes don't see what the mind don't know; it is important to address this deficit during development of intervention strategy to reduce DUP.

Thirdly, lack of knowledge about treatment efficacy, standards of treatment setting and the right process to seek help further added to delay in help-seeking at a psychiatric facility among caregivers in the current study. This current study highlighted the tendency for people to be misled by rumors or biased reporting in social media which contributes to the generation of stigma when accurate information is not made available. This finding supports evidence from the past on the development of negative views especially in young individuals which led to stigma arising from rumors or biased information about mental illness in the absence of accurate information (33–35).

In this current study, stigma was found to be an equally important contributor to delay in professional help-seeking behavior in schizophrenia. Negative view on mental illness and its treatment coming from society is often internalized by the patients and their caregivers which hinder help-seeking for fear of being disgraced, disrespected, isolated and discriminated against. Stigma alone in the presence of right knowledge about mental illness was still a hindrance to professional help-seeking among the caregivers in this study. Stigma combined with inadequate knowledge was an even worse situation which lengthened the help-seeking process. Nevertheless, this relationship between knowledge, stigma and help-seeking is a dynamic process i.e. improving knowledge facilitates removal of stigma and encourages help-seeking. For example, stigma and avoidance of help-seeking in caregivers arising from unfavorable and inaccurate media reporting on psychiatric treatment setting improved when more accurate information was obtained. Inadequacy of the right knowledge being a large contributor to formation of stigma has been supported by many past studies (33, 36, 37). Improving reporting on mental illness and its treatment, especially in social media, thus should be recognized as an important intervention that could reduce stigma and facilitate professional help-seeking.

Cultural factors may also influence professional help-seeking in schizophrenia. High tolerance for behavioral abnormality and disability in Asian families often leads to symptoms being unrecognized (38). Misattribution of illness behaviors to typical adolescence, stress, lifestyle or supernatural causes, which was also observed in this current study, is another common cultural contribution to delay in help-seeking (39, 40). In Malaysia, beliefs about causes of schizophrenia vary. Although some attribute psychotic behavior to illness, many others believe that psychosis is a result of evil spirit, black magic, or spiritual related problem. This may arise from inaccurate understanding of religious stand on mental illness commonly conveyed by religious preachers and/or unconscious shifting of responsibility from another set of beliefs that mental illness is a sign of weakness and flaw. Schizophrenia may also be regarded as retribution for past wrongdoing of an individual (41). This does not only influence the choice of initial help sought but the stigma that follows might cause families to conceal the matter and delay help-seeking. Previous studies done in other countries have consistent results with this study (21, 42–44). Hence, addressing these cultural issues while developing intervention for promoting professional help-seeking behavior is important.

Findings from this current study could serve as important material for developing educational material about schizophrenia in developing countries such as Malaysia. In addition, the qualitative method employed in this study has an added advantage in capturing some of the patient or family-related factors that could be a limitation in quantitative study. Our initial objective was to include participants from patients with schizophrenia and their caregivers as study participants in order to gather richer and more complete data on seeking first psychiatric treatment. However, preliminary analysis of the result in the In-Depth Interviews (IDI) with up to 6 patients showed that all patients lost their reality testing during their acute episode at the onset of their illness and had to rely on family members totally to seek help. A patient recalled “I can't differentiate between real and hallucinatory voices when I was ill, the voices are the same” (Participant 1, 2018). Another patient recalled “I don't know doctor, my mind was so muddled up, I can't even recall now what happened at that time” (Participant 3, 2018). All the participants recalled that decision for initial treatment was not decided by themselves. Further IDI session was halted as it was learned at this stage that caregivers were the main determinant for the initial help-seeking decisions and actions. Caregivers play an important role in determining how promptly a patient is brought forward for psychiatric treatment in early psychosis (15).

The results of our study, however, should be interpreted with certain caveats. Caregivers from the rural areas were not represented in this study as the research was conducted in a centre for urban population. Therefore, the findings may not be generalized to the rural population. In this study, data collection on the patient group was terminated before saturation point was reached as all the participants in the first six interview session recalled that decision for initial treatment was not decided by themselves due to impaired reality testing and object relation. Though not specifically done for first episode psychosis, literature does describe that reality testing and object relation is impaired when a person undergoes acute phase of psychosis (45, 46). Hence, with the objective of promoting professional help-seeking behavior during first episode psychosis in mind, we found that it is more relevant to focus on caregivers rather than patients themselves when they are in the acute psychosis phase. However, this does not mean education for schizophrenia patients is less important as patient's reality testing and object relation improve when they are clinically stable. Psycho-education in both groups, the patients and their caregivers, does promote treatment adherence and relapse prevention.

## Conclusion

Knowledge about and stigma attached to mental illness play an important role in professional help-seeking behavior among caregivers of people with schizophrenia. High level of right knowledge on schizophrenia and its treatment and low level of stigma attached to it better encourages professional help-seeking behavior. However, stigma can undermine the impact of knowledge if not addressed well. Intervention strategies for promoting help-seeking behavior among patients during FEP should simultaneously focus on improving both knowledge about schizophrenia and the stigma attached to it.

## Data Availability Statement

All datasets generated for this study are included in the article/supplementary material.

## Ethics Statement

This research study had obtained approval from Unitversiti Kebangsaan Malaysia Medical Centre Ethics Committee. Written informed consent was provided by all participants.

## Author Contributions

DW designed the research instruments, completed all data entry and data analysis, and wrote the manuscript as part of his master's research. MM is the principal supervisor of this project with co-supervision from ST, TM, and SA. DW, ST, and MM contributed to patients' recruitment, data collection, and interpretation of data. All authors were involved in the intellectual work for the article including generating research ideas, planning for the research, drawing up the manuscript, reading and approving the final manuscript.

## Conflict of Interest

The authors declare that the research was conducted in the absence of any commercial or financial relationships that could be construed as a potential conflict of interest.
